# Supplementary material for: Carbazole and Diketopyrrolopyrrole-Based D-A π-Conjugated Oligomers Accessed via Direct C–H Arylation for Opto-Electronic Property and Performance Study
Source: Molecules. 2022 Dec 18;27(24):9031. doi: 10.3390/molecules27249031 (PMC9781591; doi:10.3390/molecules27249031)
Supplement: Supplementary file 1 [file molecules-27-09031-s001.zip › molecules-2090554-supplementary.pdf]

Carbazole and Diketopyrrolopyrrole-based D-A π-Conjugated Oligomers Accessed *via* Direct C–H Arylation for Opto-electronic Property and Performance Study

Xiafeng Zhang[1] †, Lingwei Feng[2] †, Kai Zhang[2,] * and Shi-Yong Liu[1,] *

[1] Jiangxi Provincial Key Laboratory of Functional Molecular Materials Chemistry, College of Materials, Metallurgical and Chemistry, Jiangxi University of Science and Technology, Ganzhou 341000, China.

[2] School of Materials Science and Engineering, South China University of Technology, Guangzhou 510000, China

* Correspondence: mszhangk@scut.edu.cn (K.Z.); chelsy@zju.edu.cn (S.-Y.L.)

† X. Z. and L. F. contribute equally to this work.

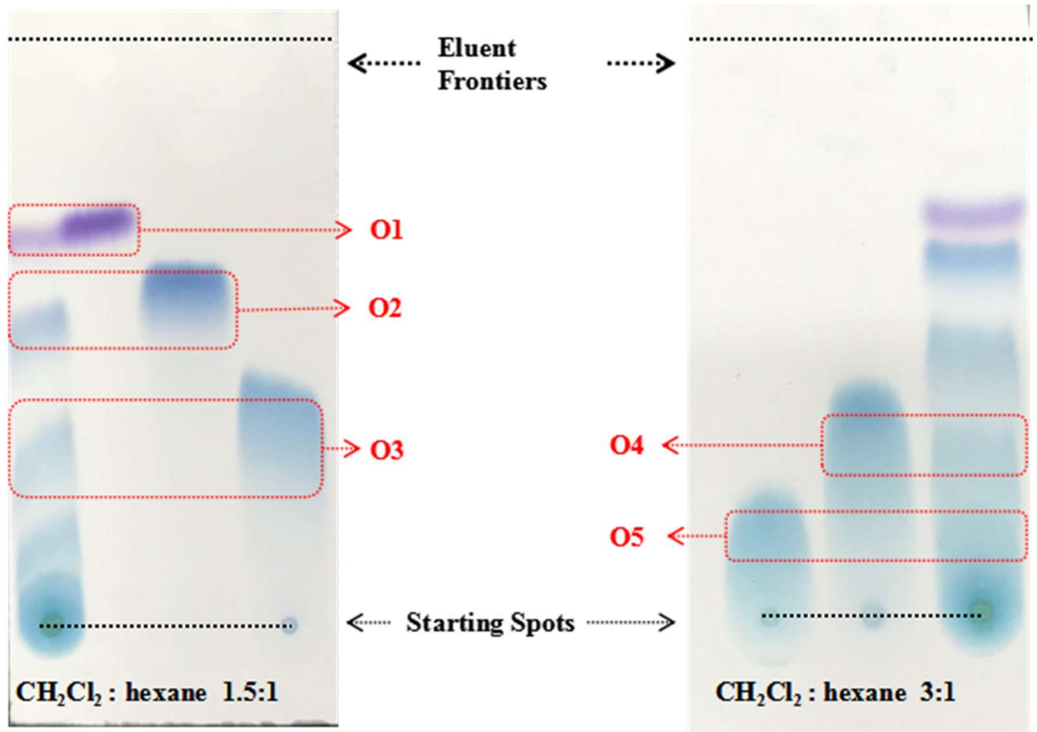

Figure S1. TLC analysis of the direct arylated coupling between DPP and Cz in molar ratio of 1.5/1 using CH_2Cl_2 : hexane (1.5:1 and 2:1 respectively, v/v) as eluent . The starting spots on each TLC plate involved the reaction mixture and the corresponding purified oligomers.

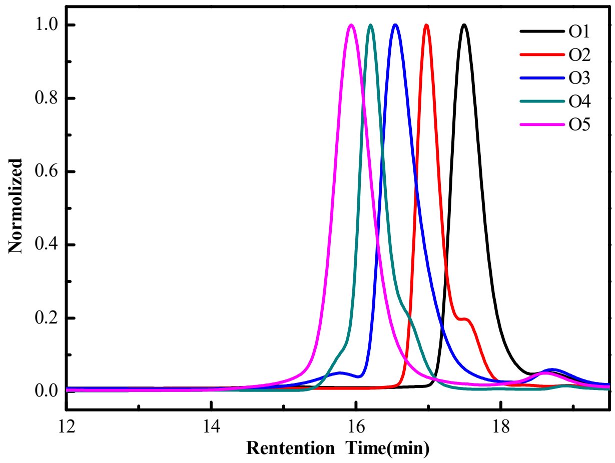

Figure S2. GPC profiles of oligomers **O**s1~5.

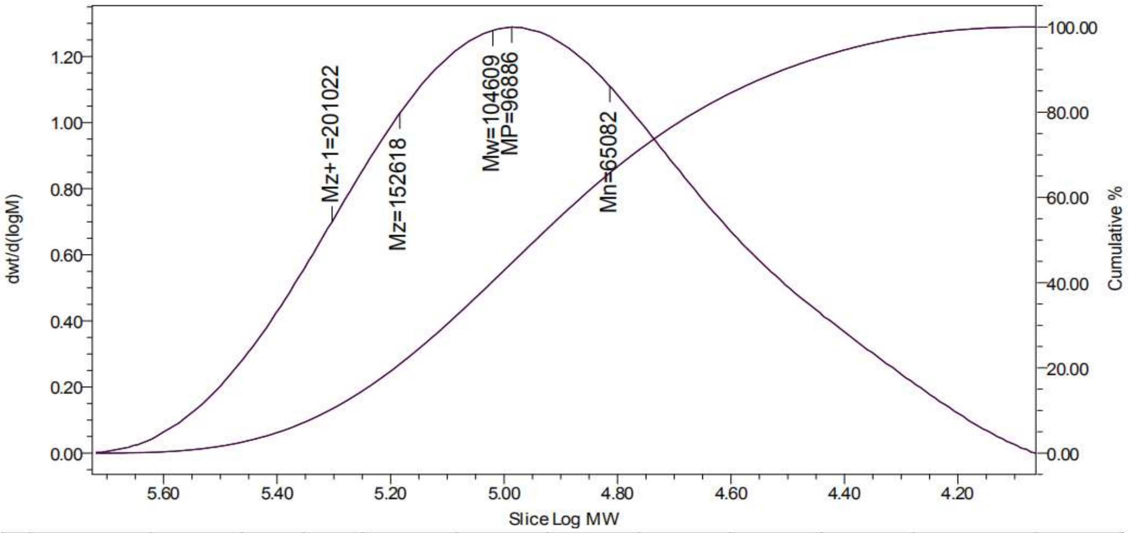

	Distribution Name	Mv (Daltons)	K (dl/g)	alpha	Intrinsic Viscosity (dl/g)	Mn (Daltons)	Mw (Daltons)	MP (Daltons)	Mz (Daltons)	Mz+1 (Daltons)	Polydispersity	Mz/Mw
1						65082	104609	96886	152618	201022	1.607330	1.458940

Figure S3. GPC profile of **P**1 and the corresponding list of the obtained data.

Table S1. Retention time in GPC, and GPC/Maldi-Tof/NMR molecular weights for **O**1~5.

	**O1**	**O2**	**O3**	**O4**	**O5**
Retention time	17.49	16.95	16.54	16.20	15.94
Mn	2150	3123	4384	5257	6357
Mw	1356	2592	3993	4969	6276
PDI	1.05	1.07	1.07	1.06	1.07
MW by maldi-tof or NMR	1450	2377	3302	4231	5162

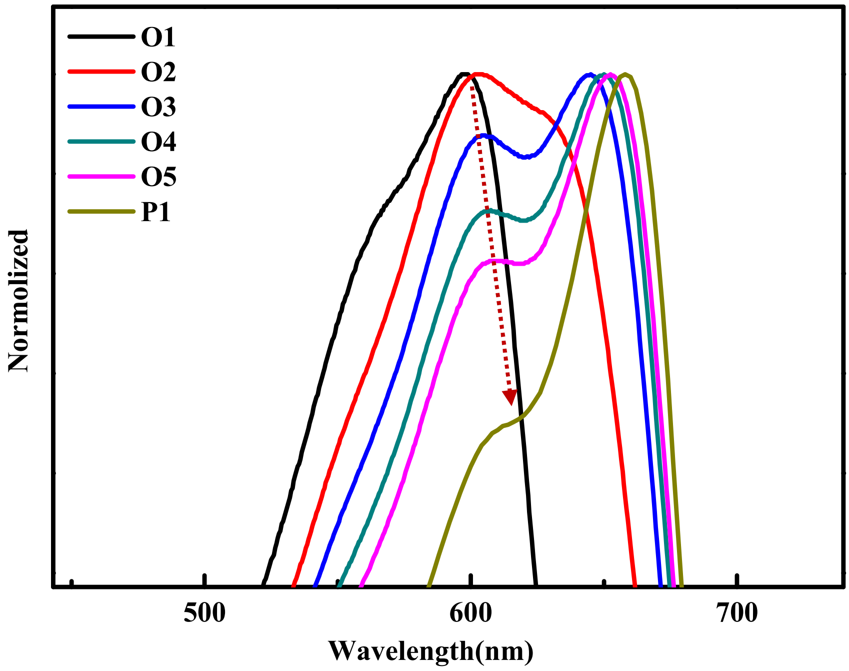

Figure S4. Partial enlargement of the absorption peaks of Uv-vis spectra of **O**s1~5 and **P**1.

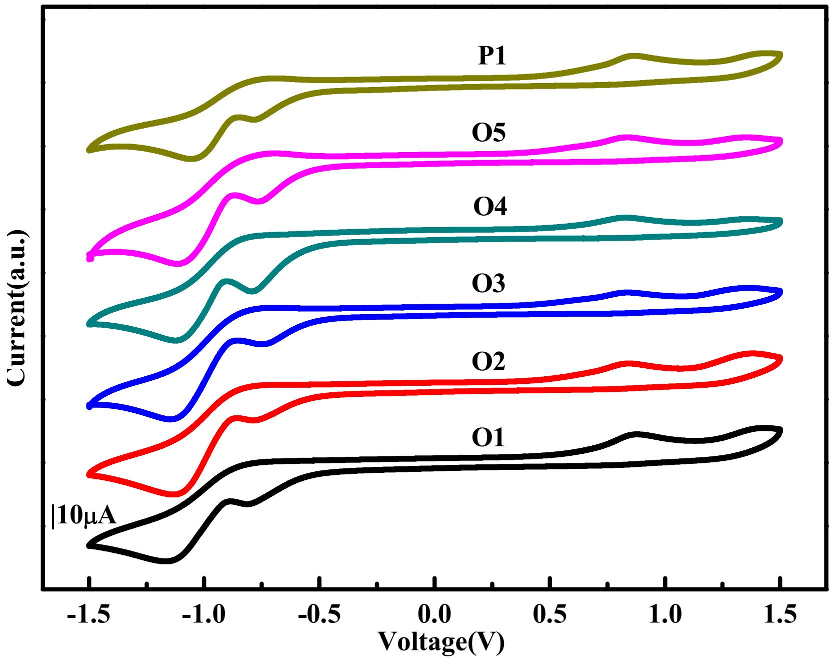

Figure S5. CV curves of oligomers Os1~5 and polymer P1.

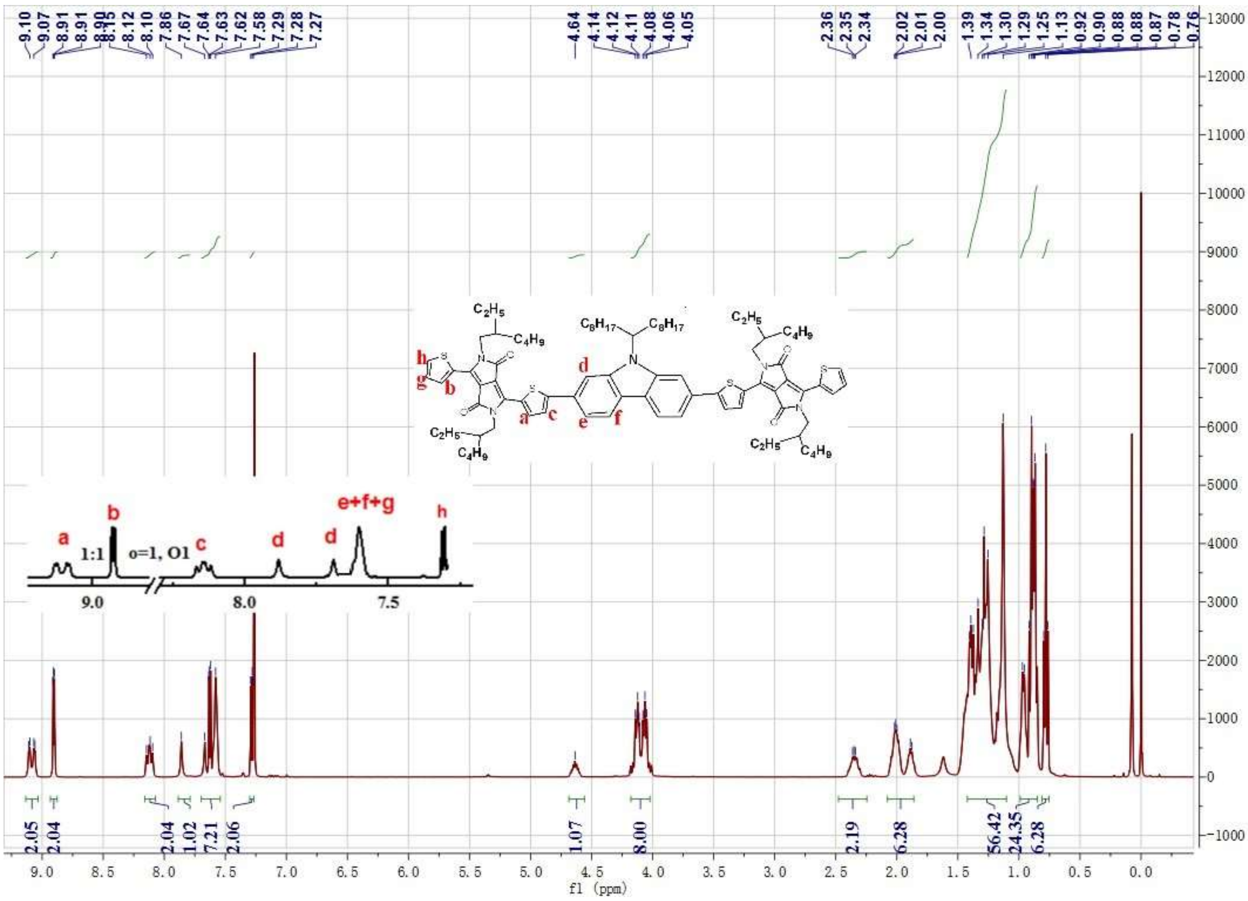

Figure S6 ^{1}H NMR spectra of **O**1 in $CDCl_3$.

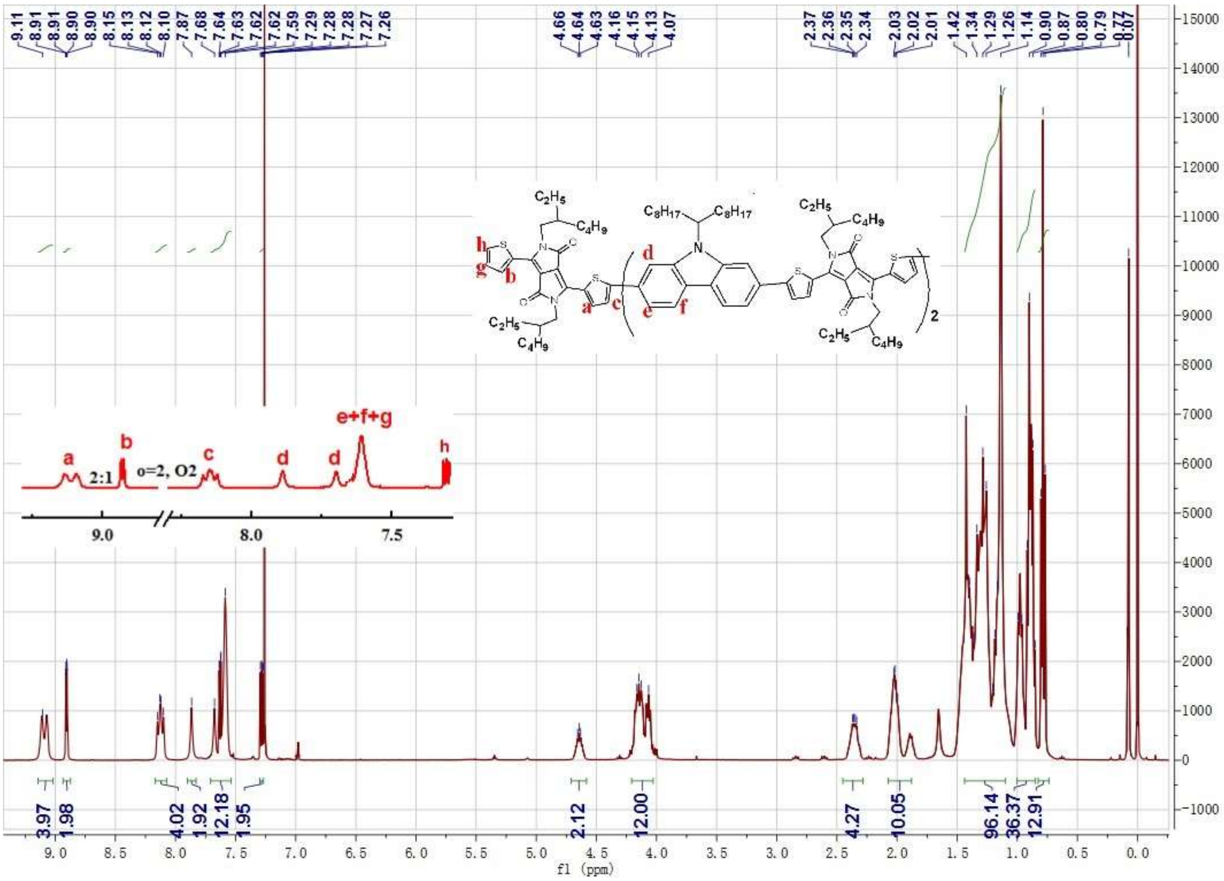

Figure S7. ^{1}H NMR spectra of **O**2 in $CDCl_3$.

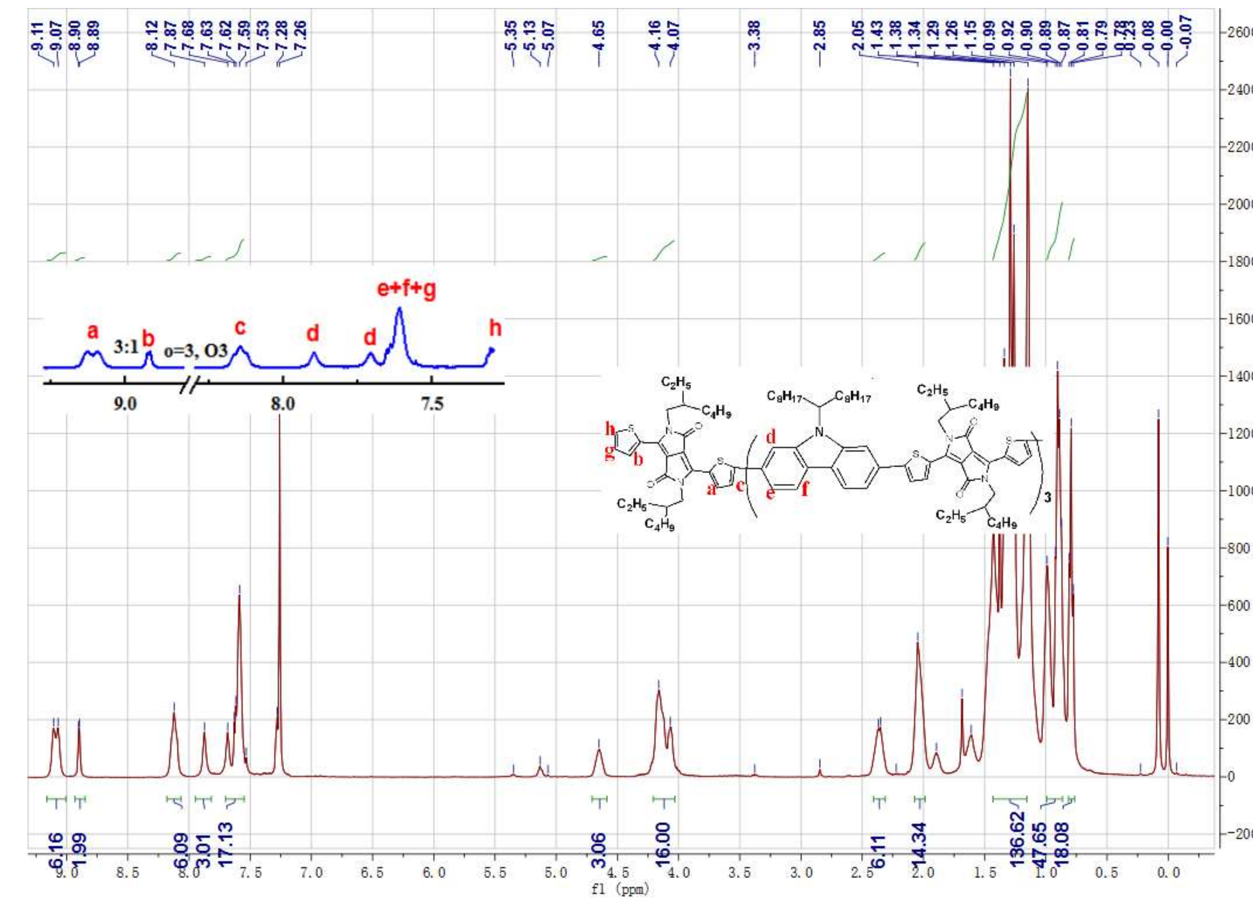

Figure S8 ^{1}H NMR spectra of **O**3 in $CDCl_3$.

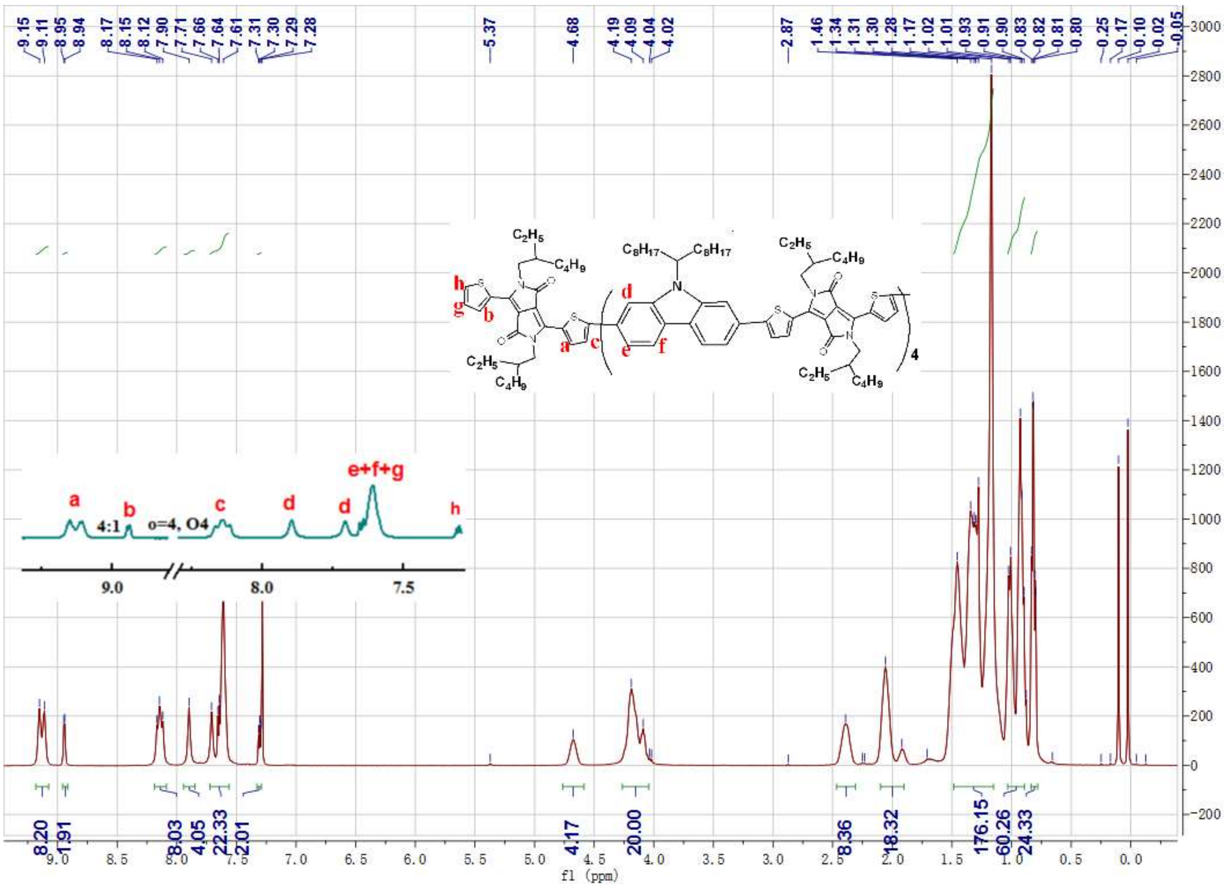

Figure S9. ^{1}H NMR spectra of **O**4 in $CDCl_3$.

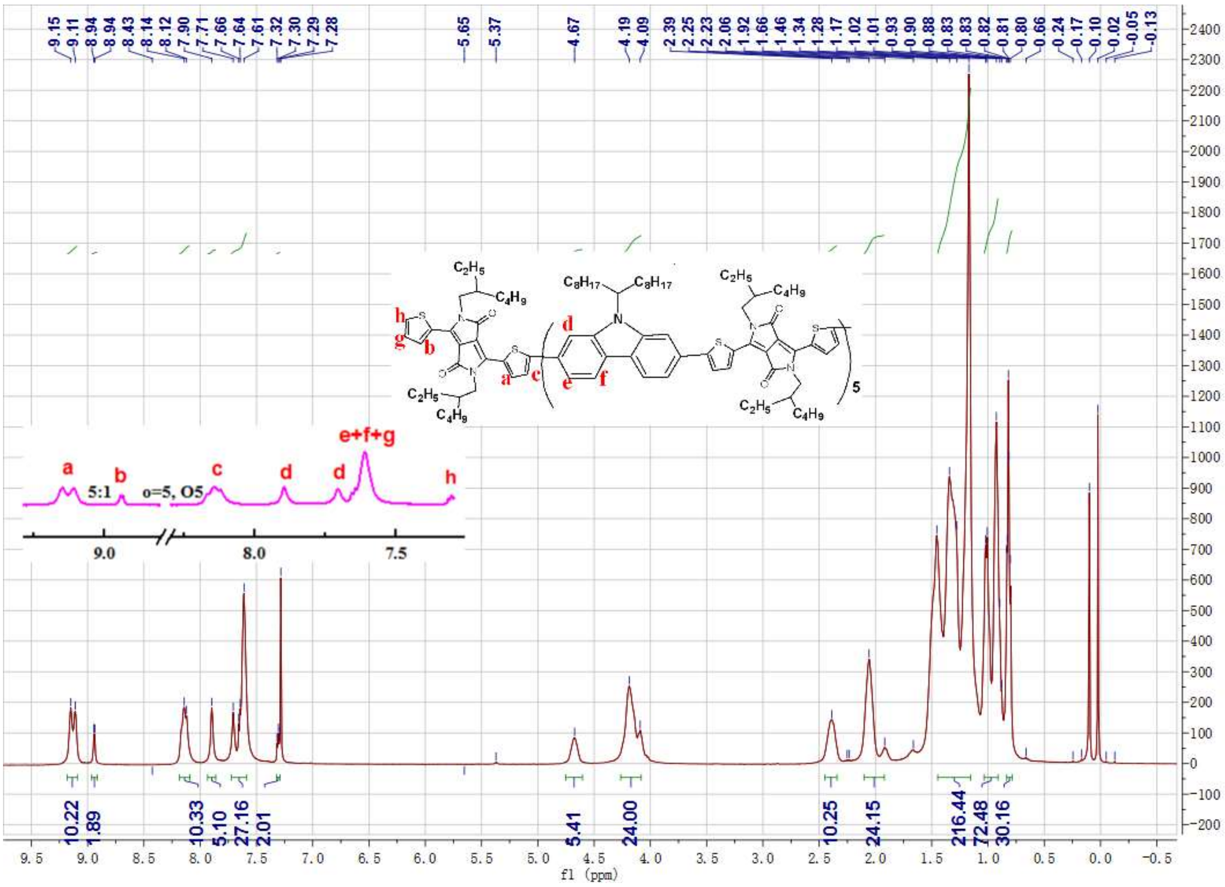

Figure S10. ^{1}H NMR spectra of **O**5 in $CDCl_3$.

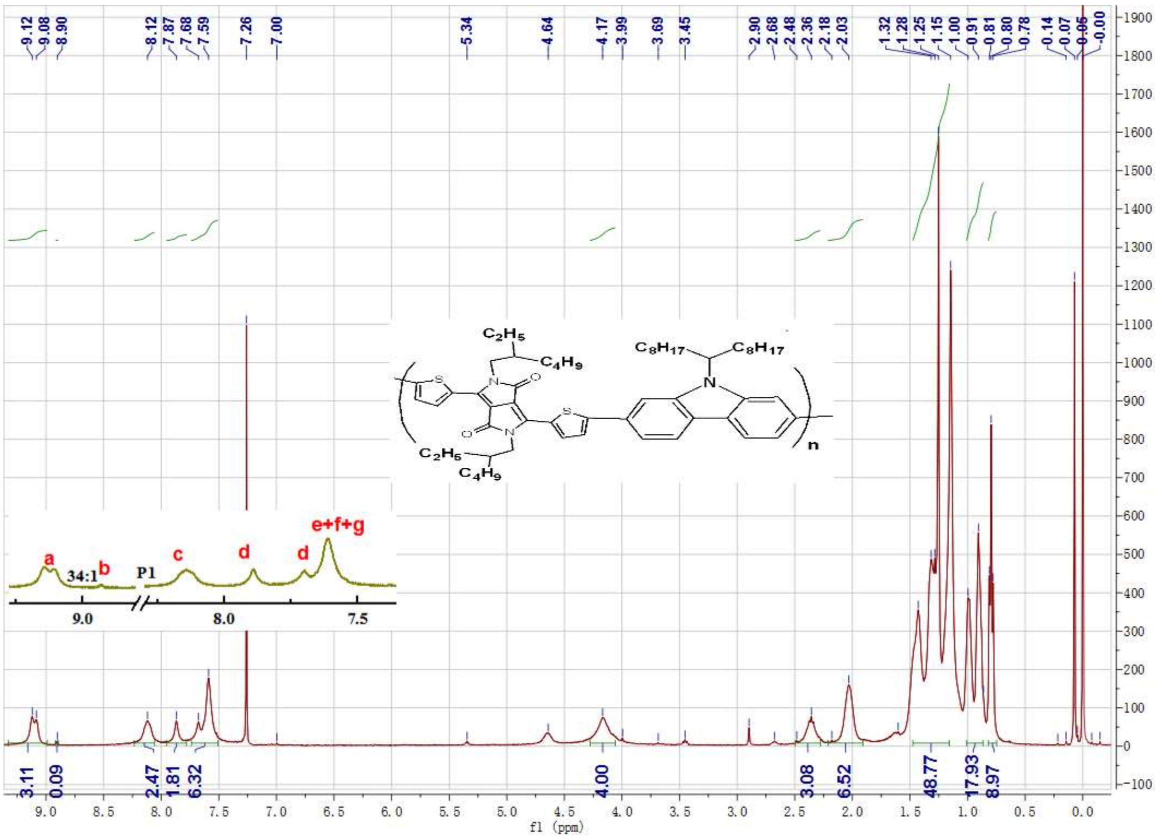

Figure S11. ^{1}H NMR spectra of **P1** $CDCl_3$.

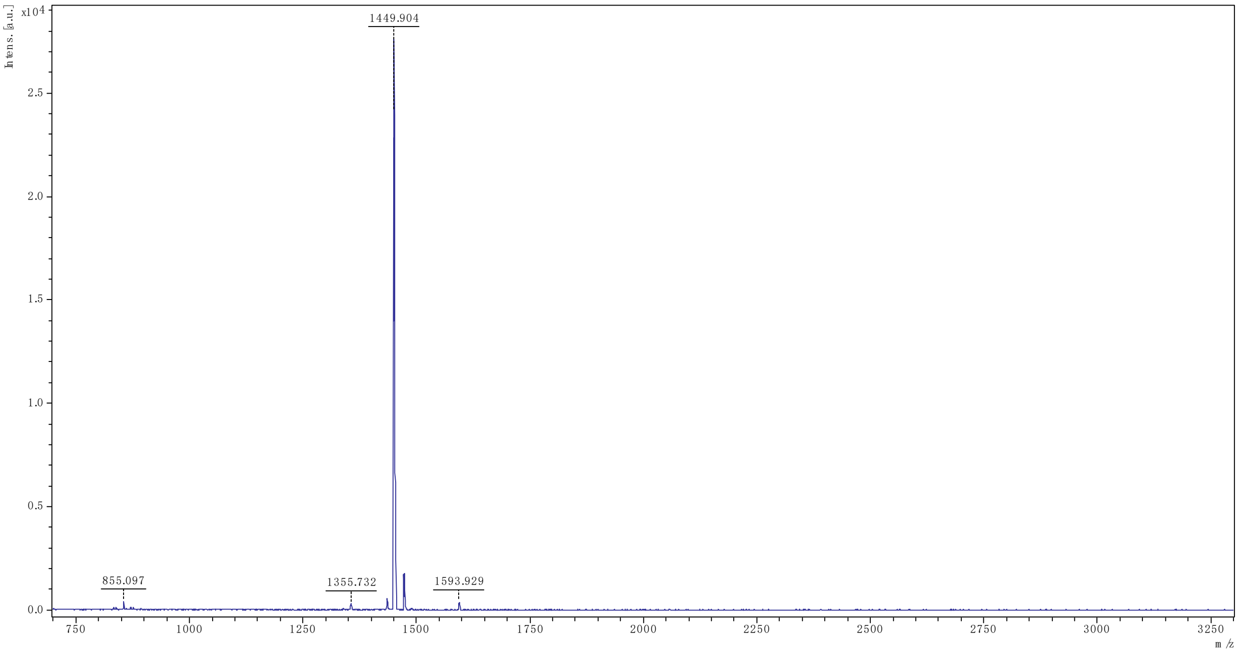

Figure S12. MALDI-TOF MS of **O**1.

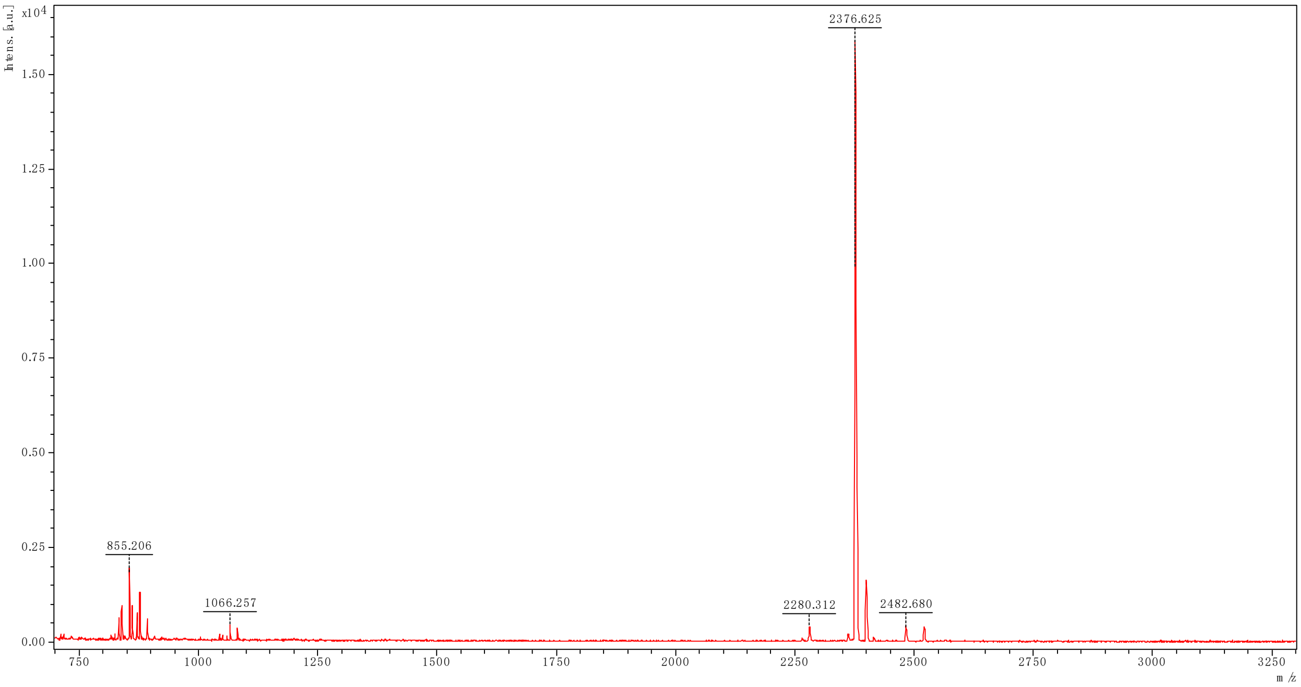

Figure S13. MALDI-TOF MS of **O**2.

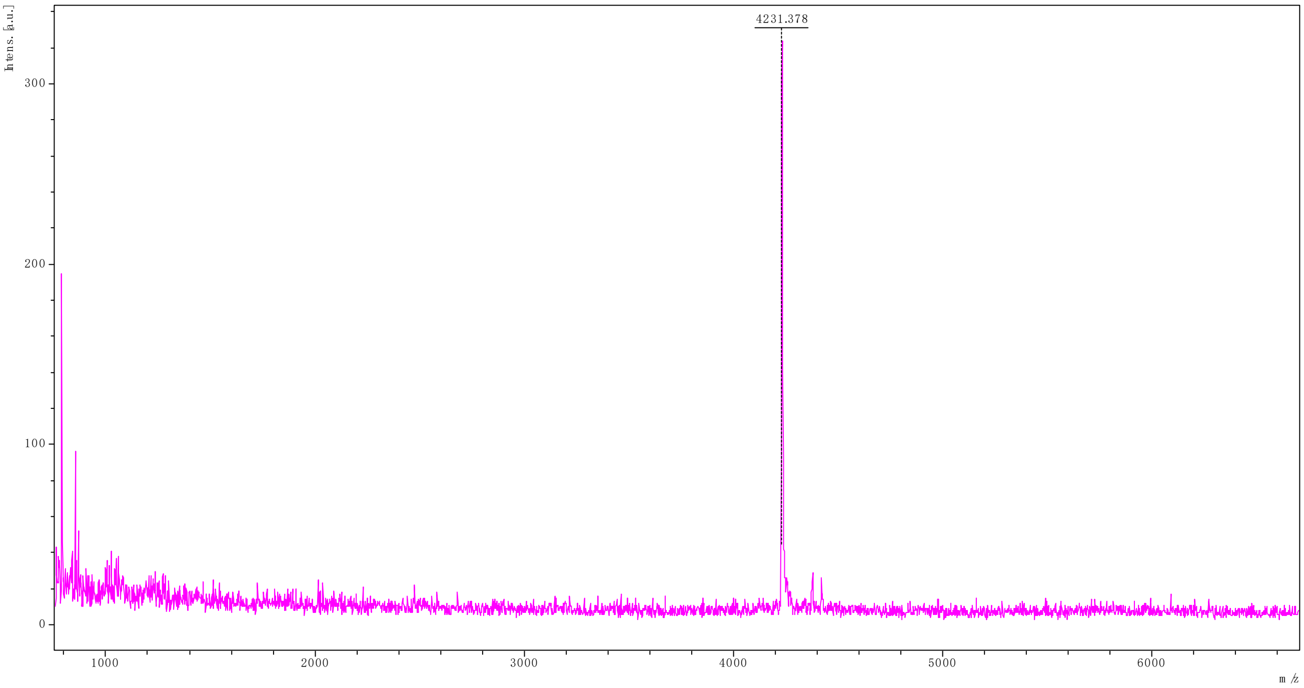

Figure S14. MALDI-TOF MS of **O**3.

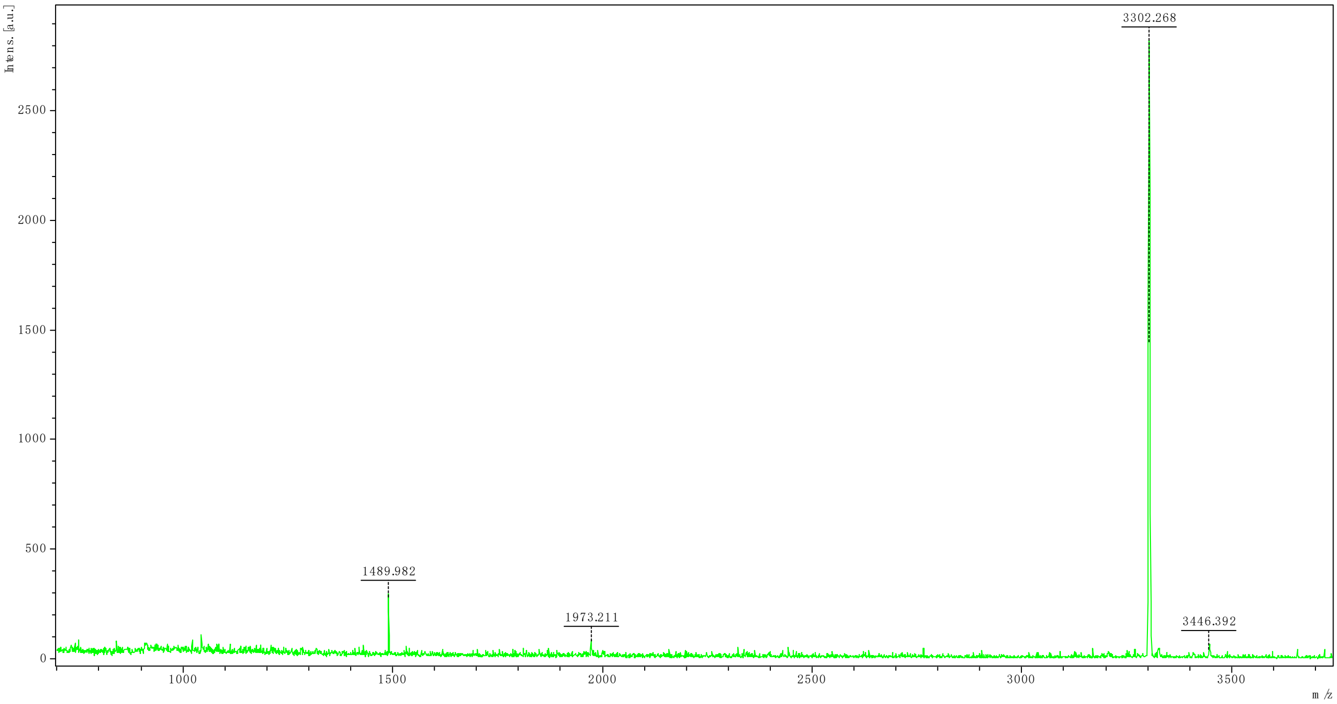

Figure S15. MALDI-TOF MS of **O**4.

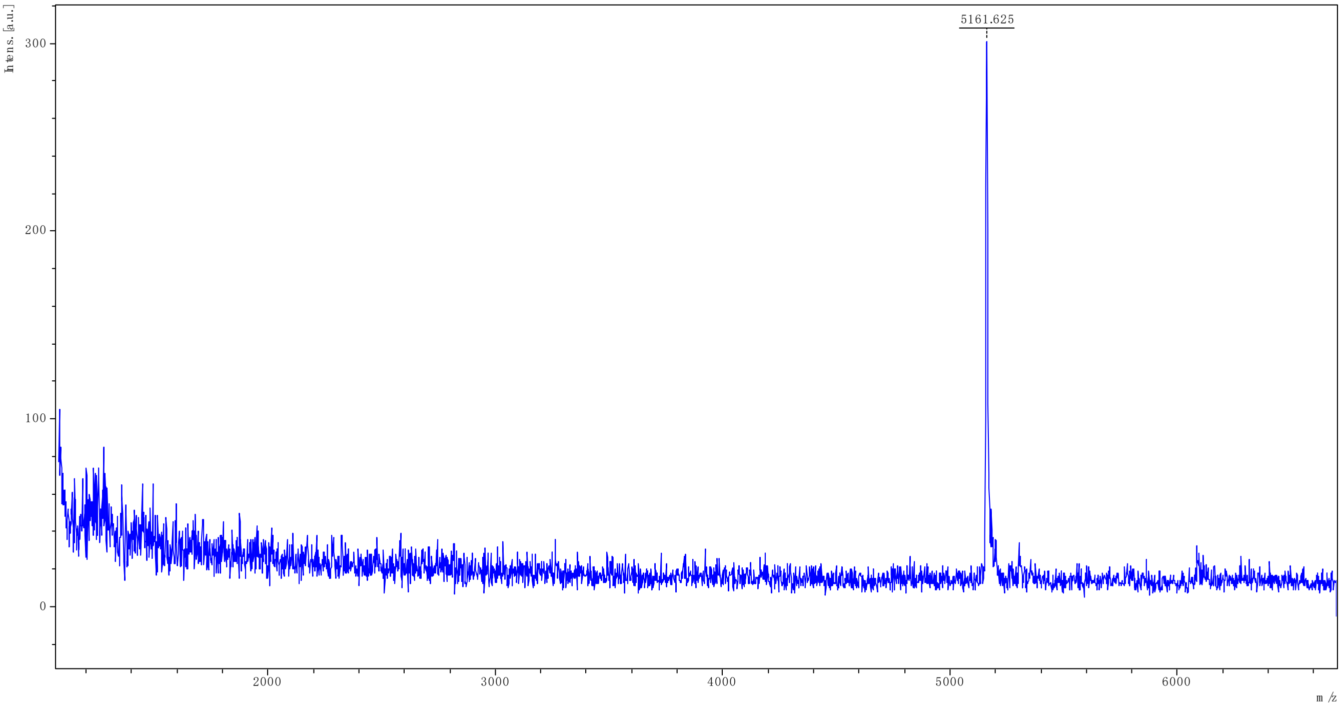

Figure S16. MALDI-TOF MS of **O**5.
